# Calpain-1 Mediated Disorder of Pyrophosphate Metabolism Contributes to Vascular Calcification Induced by oxLDL

**DOI:** 10.1371/journal.pone.0129128

**Published:** 2015-06-05

**Authors:** Futian Tang, Erqing Chan, Meili Lu, Xiaowen Zhang, Chunmei Dai, Meng Mei, Suping Zhang, Hongxin Wang, Qing Song

**Affiliations:** 1 Key Laboratory of Cardio- and Cerebro-vascular Drug Research of Liaoning Province, Drug Research Institute, Liaoning Medical University, Jinzhou, China; 2 Cerebrovascular Diseases Center of Gansu Provincial Hospital, Lanzhou, China; 3 Guangzhou Institute of Sports Science, Guangzhou, China; 4 Guangzhou Vocational and Technical College, Guangzhou, China; University of California, Los Angeles, UNITED STATES

## Abstract

We previously reported that oxidized low density lipoprotein (oxLDL) accelerated the calcification in aorta of rats and rat vascular smooth muscle cells (RVSMCs). However, the molecular mechanism underlying the acceleration remains poorly understood. The present study aimed to investigate the role of calpain-1, Ca^2+^-sensitive intracellular cysteine proteases, in the vascular calcification of rats treated with both high dose of vitamin D_2_ and high cholesterol diet. The results showed that calpain activity significantly increased in calcified aortic tissue of rats and RVSMCs treated with oxLDL. Specific calpain inhibitor I (CAI, 0.5mg/kg, intraperitoneal) inhibited the vascular calcification in rats with hypercholesterolemia accompanied by the increase in the level of extracellular inorganic pyrophosphate (PPi), the endogenous inhibitor of vascular calcification. In addition, CAI increased the content of adenosine triphosphate (ATP), decreased the activity, mRNA and protein expression of alkaline phosphatase (ALP) and reduced the production of superoxide anion in calcified aortic tissue. CAI also increased the activity of ATP synthase as well as protein expression of ATP5D, δ subunit of ATP synthase. In the *in vitro* study, suppression of calpain-1 using siRNA assay inhibited the calcium deposition, increased the levels of PPi and ATP, improved the activity of ATP synthase as well as protein expression of ATP5D in RVSMCs treated with oxLDL. Calpain-1 suppression also decreased the activity, mRNA and protein expression of ALP and reduced the mitochondrial ROS (Mito-ROS) production in RVSMCs. However, mito-TEMPO, the mitochondria-targeted ROS scavenger, reduced the calcium deposition, increased the PPi in culture medium, decreased the activity, mRNA and protein expression of ALP in RVSMCs treated with oxLDL. Taken together, the results suggested that calpain-1 activation plays critical role in vascular calcification caused by oxLDL, which might be mediated by PPi metabolism disorder. The results also implied that Mito-ROS might contribute to the PPi metabolism disorder through regulation of the activity and expression of ALP.

## Introduction

Vascular calcification characterized by calcium-phosphate deposition (CPD) in distinct layers of the aortic wall is an important risk factor for cardiovascular events due to the decreased aortic compliance and elastic recoil [1–4]. Medial calcification occurs within the elastin region of arteries and is almost exclusively associated with vascular smooth muscle cells (VSMCs) [5]. Several studies showed that extracellular inorganic pyrophosphate (PPi) directly inhibits *in vitro* and *in vivo* CPD and is therefore an important endogenous inhibitor of vascular calcification [6–8]. Degradation of PPi is catalyzed by tissue-nonspecific alkaline phosphatase (ALP), which hydrolyzes PPi to Pi. Importantly, calcification in *ex vivo* cultured rat aorta is induced by ALP and is prevented by ALP inhibitors [9]. ALP is up-regulated in the aortas of uremic rats, which results in increased hydrolysis of PPi and vascular calcification [10]. The ectoenzyme nucleotide pyrophosphatase/phosphodiesterase-1 (Enpp1) is the main enzyme involved in PPi synthesis [8]. Lack of eNPP1 results in extensive and fatal arterial calcification in mice and children [11, 12]. The substrate for eNPP1 is ATP, which accumulates in the extracellular matrix via the action of transporters [13], such as the multiple-pass transmembrane protein ANK [14]. ATP is synthesized through catalysis of ATP synthase such as ATP5D, δ subunit of ATP synthase, in mitochondria. Incapability of ATP synthesis was shown to be responsible for the VSMCs calcification [15, 16]. Therefore, correction of the imbalance between PPi production and degradation might be the potentially therapeutic target for vascular calcification and the related the diseases.

Calpains, the Ca^2+^-sensitive intracellular cysteine proteases, tightly regulate their respective substrates through limited proteolytic cleavage [17, 18]. Calpains recognize various intracellular substrate molecules, thereby controlling cellular activities. Numerous *in vivo* and *in vitro* experiments employing genetic and pharmacological approaches have focused on the roles of calpains in cardiovascular diseases [19–23]. Calpain-1 was increased in calcified aortic wall of rats with ageing at levels of transcripts, protein, and activity [24]. Over-expression of calpain-1 induced VSMC calcification, which were antagonized by over-expression of calpastatin, a specific endogenous inhibitor of calpain-1 [25]. These results suggested that calpain-1 plays a key role in vascular calcification. Several mechanisms by which calpain-1 gets involved in the vascular calcification were reported. Over-expression of calpain-1 was found to decrease the levels of osteopontin and osteonectin, the anti-calcification factors and to increase the expressions of matrix metalloproteinase 2 (MMP2), collagen I, II, and III, the pro-calcification factors. However, the precise mechanism underlying the involvement of calpain-1 in vascular calcification remains unknown. Based on the fact that calpain-1 has been shown to selectively proteolyze enzyme proteins [19], we hypothesized that calpain-1 proteolyzes ATP5D and reduces ATP production, which causes the disorders of PPi metabolism, subsequently leading to the calcification.

The *in vitro* studies indicated that oxidized low density lipoprotein (oxLDL) enhances the calcification of VSMCs by stimulating the osteoblastic differentiation of vascular cells [26, 27]. We reported in the previous *in vivo* and *in vitro* studies that oxLDL enhanced the vascular calcification through oxidative stress and up-regulation of ALP expression [28]. However, the exact mechanism underlying the enhancement is not yet clear. Based on the reports showing that oxLDL induces calpain activation [29] and up-regulates the ALP expression in VSMCs [28], the present *in vivo* and *in vitro* studies were designed to investigate the roles of calpain-1 in the vascular calcification induced by oxLDL with focus on the PPi metabolism. The results showed that calpain-1 mediates vascular calcification caused by oxLDL by inducing the disorders of PPi metabolism.

## Materials and Methods

### Chemicals and reagents

Calpain inhibitor I (N-acetyl-leu-leu-norleucinal, CAI) was purchased from Santa Cruz Biotechnology (sc-29119, MW: 383.5). 100mg of CAI was dissolved in 1ml of DMSO, which was the stock solution with concentration of 100mg/ml. The stock solution was diluted 1000 times with sterilized distilled water before use and the final concentration of CAI was 0.1mg/ml. Dihydro-ethidium bromide (DHE), Vitamin D_2_, β-glycerophosphate, phosphoenolpyruvate, pyruvate kinase, lactate dehydrogenase, adenosine 5′-phosphosulfate and ATP-sulfurylase purchased from Sigma (St. Louis, MO).

### Animals and vascular calcification procedures

This investigation was carried out in strict accordance with the recommendations in the Guide for the Care and Use of Laboratory Animals of the National Institutes of Health. The study was approved by the Committee on the Ethics of Animal Experiments of the Liaoning Medical University, China (Permit Number LNMU-2014-128). Male Sprague—Dawley (SD) rats (220±20g) aged 6-weeks were purchased from the Experimental Animal Center, Liaoning Medical University of China. Rats were randomly divided into Control, high cholesterol diet (HCD) and CAI groups, with 8 animals in each group. The preparation for vascular calcification model of rats was described previously [28, 30]. Briefly, all rats were orally administered with 300,000 IU/kg/day vitamin D_2_ for the first 4 consecutive days only followed by consuming standard chow in control group and HCD in HCD group and CAI groups for 8 weeks. HCD was composed of standard chow (94.3%), cholesterol (2%), lard (3%), cholic acid (0.5%), propylthiouracil (0.2%). On the first day of the vitamin D_2_ administration, rats in CAI group were given 5ml/kg of 0.1mg/ml CAI (0.5mg/kg, once daily) by intraperitoneal injection, and rats in control and HCD groups were given vehicle by intraperitoneal injection. After the fasting rats (12h) were anesthetized with 30 mg/kg pentobarbital sodium at the end of the experiment, blood was collected from abdominal aorta for serum separation. Thoracic abdominal aorta was isolated and the connected tissue carefully removed followed by storage at -70°C until use. Aorta arch was removed and fixed in 10% formalin.

### Morphological evaluation of vascular calcification

Cross sections of aortic arch were stained with von Kossa kit (Nanjing Jiancheng Bioengineering Company, China) for visualization of the vascular calcification as described previously [28]. Briefly, aortic arch was fixed in 10% formalin followed by dehydration and embedding in paraffin. Six-micrometer thick sections were deparaffinized and dehydrated before being immersed in a light-protected 5% AgNO_3_ for 30 min and then immersed in a solution of 5% sodium thiosulfate for 2 min followed by counterstaining with eosin. Images were taken using microscope (Leica DMI 3000B, Germany) and analyzed with LAS Software (V4.3) (Leica, Germany).

### Calcium content determination in aortic tissue

The calcium content in aortic tissue was determined as described previously [28]. Briefly, the aortic tissue was digested in HNO_3_ and then dried in an oven and dissolved with the blank solution (27 nmol/L KCl, 27 μmol/L LaCl3 in de-ionized water). The calcium content was measured by an atomic absorption spectrophotometer at 422.7 nm (Shimadzu, AA-670, Kyoto). Calcium content was expressed by mg/g dry tissue.

### Serum oxLDL determination

Serum content of oxLDL was measured by enzyme linked immunosorbent assay (ELISA) using kit (Mebio Company, China) as described previously [31].

### RVSMCs culture

Rat vascular smooth muscle cells (RVSMCs) were separated from rat aorta and cultured as described previously [28]. Immunocytochemical examination showed positive staining in all cells for α-smooth muscle actins.

### Measurements of superoxide anion in aorta and ROS in mitochondria of RVSMCs

Superoxide anion in aortic tissue was measured using dihydroethidium (DHE) as described previously [28]. Mito-SOX red (Boyetime Institute of Biotechnology, China), the specific indicator for ROS in mitochondria, was used to measure the ROS in mitochondria of RVSMCs as described previously [32]. Images were taken using microscope (Leica DMI 3000B, Germany) and analyzed with LAS Software (V4.3) (Leica, Germany).

### Transfection of calpain-1 and calpain-2 siRNA

Small-interfering RNA (siRNA) mediated gene silencing of calpain-1 and calpain-2 was carried out by transfecting RVSMCs with rat calpain-1 and calpain-2 siRNA (Santa Cruz Biotechnology), using a scrambled siRNA as a transfection negative control as described previously [33]. Briefly, RVSMCs (5 to 10 generations) were allocated into negative control and siRNA interference groups. Cells were seeded in a 24-well plate at a density of 2×10^5^ and incubated for 12 h. Then, cultured RVSMCs were transfected using Lipofectamine-2000 (Invitrogen) according to the manufacturer’s instructions. Six hours later, the medium was replaced with DMEM medium containing 10% FCS. The effectiveness of siRNA in down-regulating calpain-1 and calpain-2 expression was confirmed by examination of protein expression and activity.

### Preparation of ox-LDL

Whole blood was obtained by venipuncture from healthy volunteers after 12 h of fasting and processed for LDL separation by sequential flotation in NaBr solution. LDL was exposed to 5 mM CuSO4 for 18 h at 37°C to get oxLDL as described previously [31]. Each volunteer was informed of the experimental procedures and signed the consent form. This study was approved by the Human Investigation Committee of the Liaoning Medical University, China (Permit Number: LNMU-2014-158).

### RVSMCs calcification and treatment

RVSMCs calcification was induced as described previously [28]. In short, normal or siRNA transfected RVSMCs were incubated in DMEM containing 20% FBS, 10 mM sodium pyruvate and 5 mM glycerophosphate supplemented with LDL (25mg/L) or oxLDL (25-100mg/L) in the presence or absence of 5μM of mito-TEMPO (Haoran Biotechnology, China) for 9 days. The medium was refreshed every 3 days and the used medium was collected and pooled together. The RVSMCs and the pooled medium were used in the following measurements.

### Von Kossa staining for calcification in RVSMCs

Calcium deposition on RVSMCs was visualized by Von Kossa staining as described previously [28]. Briefly, cell monolayers were fixed in 0.1% glutaraldehyde for 15 min at room temperature. Cells was then washed twice with ddH_2_O and incubated with 5% silver nitrate for 30 min at room temperature in the dark. Silver nitrate was removed, and the cells were rinsed twice with ddH_2_O. After being dried by air, cultures were exposed to sunlight until color development was complete. Images were taken using microscope (Leica DMI 3000B) and analyzed with LAS Software (V4.3) (Leica, Germany).

### Content of calcium deposition on RVSMCs

Content of calcium deposition on RVSMCs was detected as described previously [28]. Briefly, the cultures were decalcified with 0.6 N HCl for 24 hours. The calcium content of the HCl supernatant was determined colorimetrically by the o-cresolphthalein complexone method. After decalcification, the cultures were washed with PBS and solubilized in 0.1 N NaOH/0.1% SDS. Total protein content was measured with a comas protein assay. The calcium content of the cell layer was normalized to protein content.

### Determination of the activities of calpain, ATP synthase and ALP

Lysates of aortic tissue or RVSMCs were used for measurement of the activities of calpain, ATP synthase and ALP. Calpain activity was determined by using a fluorescence substrate N-succinyl-LLVY-AMC (Amyjet Scientific Inc, China) as described previously [19]. The fluorescence intensity at 400 nm excitation and 505 nm emission wavelengths was measured using Synergy 2 Multi-Mode Microplate Reader (BioTek, Germany). ATP synthase activity was measured using an assay coupled with pyruvate kinase, which converts ADP to ATP and produces pyruvate from phosphoenolpyruvate as described previously [34]. Briefly, lysates of aortic tissue or RVSMCs were incubated in a buffer containing (in mmol/l) 20 HEPES, 5 MgCl2, 100 KCl, 5 KCN, 2.5 phosphoenolpyruvate, and 0.2 NADH with 0.1 mg/ml pyruvate kinase and 0.1 mg/ml lactate dehydrogenase (pH 7.5–8.0). The reaction was initiated by the addition of ATP to a desired final concentration (1 mM) and followed by the decrease in NADH absorption at 340-nm wavelength. Absorbance was measured on a Biotek Synergy HT plate reader (Biotek, Winooski, VT), and protein content was assessed as described above with final values expressed as micromoles consumed per minute per milligram of protein, which was equal to nanomoles of NADH oxidized per minute per milligram of protein. ALP activity was determined using kit from Jiancheng Bioengineering Company (Nanjing, China) as described previously [28].

### mRNA expression of ALP, Enpp1 and Ank

The mRNA expression of ALP was analyzed by quantitative real time RT-PCR using the BioRad iQ5 Real Time PCR system (BioRad Company) as previously described [28]. Briefly, total RNA from aortic tissue or RVSMCs was extracted with TRIzol reagent (Invitrogen). The first strand cDNA was synthesized using AMV reverse transcriptase (TaKaRa, Dalian, China). Amplification was performed according to the instruction of SYBR Premix Ex Taq kit (TaKaRa, Dalian, China). The cDNA was denatured at 95°C for 5 seconds followed by PCR 40 cycles (95°C, 5s; 60°C, 30s). All results were repeated four independent experiments. The relative level of mRNA was calculated by the comparative C_T_ method with GAPDH mRNA as the invariant control. The sequences of the primers (Invitrogen Biotechnology, Shanghai, China) used are: 1) ALP: forward, 5'-CTA TGT CTG GAA CCG CAC TGA-3'; reverse, 5'-AGC CTT TGA GGT TTT TGG TCA-3'. 3) Enpp1: forward, 5'-GGA TTG TGC CAA TAA GGA CT-3'; reverse, 5'-CAA GAA CTG TTG CTG CTG GAG-3'. 4) Ank: forward, 5'-CAT CCC CAT CCT GTC TCT GTA-3'; reverse, 5'-ACA CGA AGA GGT TGA CAA TGG-3'. 5) GAPDH: forward, 5'-ATG TTT GTG ATG GGT GTG AAC CAG G-3'; reverse, 5'-TAG CCA TAT TCA TTG TCA TAC CAG G-3'.

### ATP and PPi quantification

PPi was measured with an enzyme-linked bioluminescence assay as described previously [7] in which PPi reacts with adenosine 5′-phosphosulfate in the presence of ATP-sulfurylase to generate ATP. For each sample, the blank (reaction without ATP sulfurylase) was subtracted to obtain the true PPi amount. ATP was measured by a coupled luciferin/luciferase reaction with an ATP determination kit (Invitrogen) using Synergy 2 Multi-Mode Microplate Reader (BioTek, Germany).

### Protein expressions of ATP5D, ALP, calpain-1 and calpain-2

Protein expressions of ATP5D, ALP, calpain-1 and calpain-2 in aortic tissue and/or RVSMCs were determined by Western blot using GAPDH as an internal standard, as described previously [28]. All antibodies were the products of Santa Cruz Biotechnology.

### Statistical analysis

Results are presented as the mean±S.D. for each group. Statistical analysis was performed by one-way ANOVA and the Student-Newman-Keuls test using SPSS 16.0 software; a value of P<0.05 was considered statistically significant.

## Results

### Calpain activity increased in aortic tissue of rats with hypercholesterolemia and in RVSMCs treated with oxLDL

**Fig 1:**
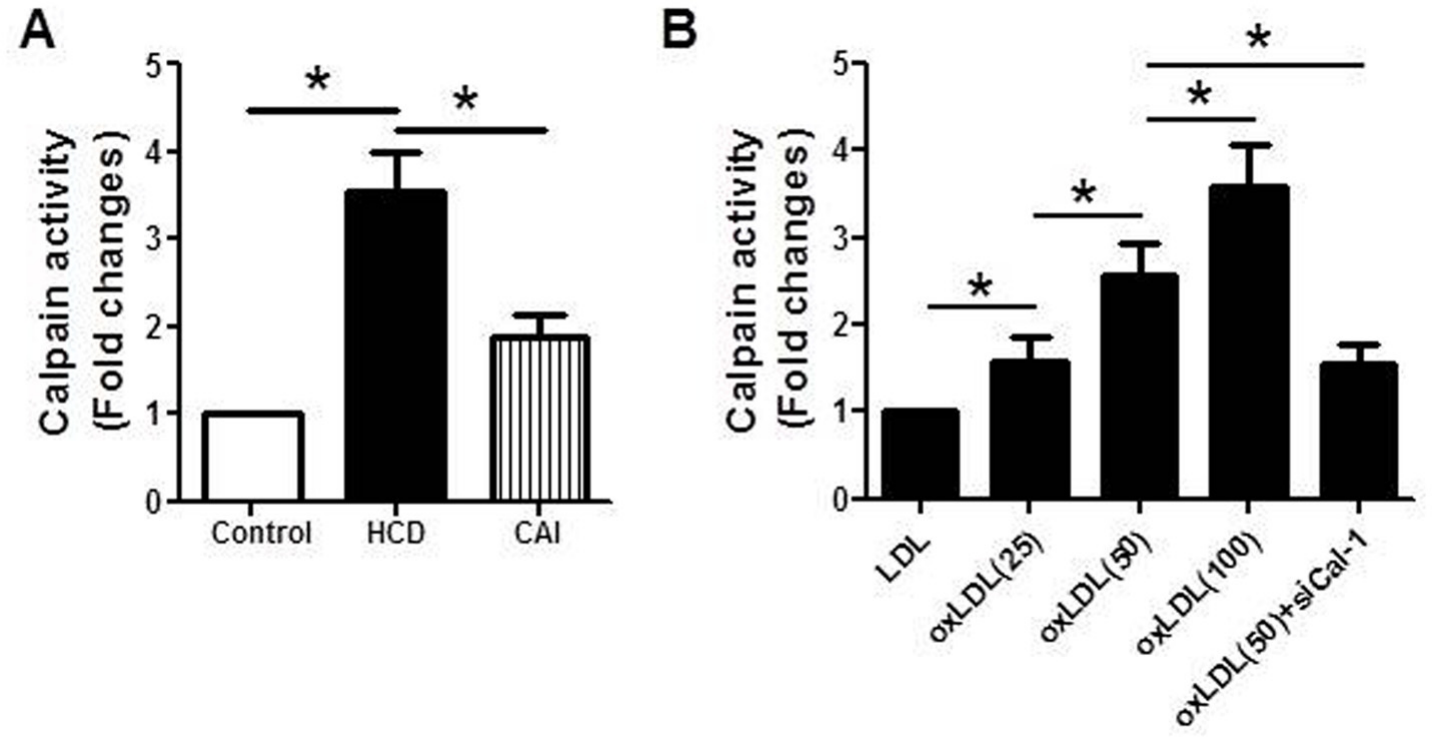
Calpain activity increased in the calcified aorta of rats and RVSMCs, which was inhibited by CAI and calpain-1 siRNA respectively. Rats were treated with high dose of vitamin D_2_ (Control) with or without high cholesterol diet (HCD) in the presence or absence of CAI. RVSMCs were treated with or without oxLDL (25-100mg/L) in the presence or absence of calpain-1 siRNA. The parameters were measured as described in Materials and Methods. A: Calpain activity in aortic tissue of rats; B: Calpain activity in RVSMCs. Results were expressed as mean±S.D. n = 8 for A and n = 4 for B. *: *P*<0.05 was considered statistically significant.

We previously reported that oxLDL enhanced the vascular calcification induced in rats with high dose of vitamin D_2_ and accelerated the calcium deposition in RVSMCs [28]. The present study showed that calpain activity in the aortic tissue of rats treated with both high dose of vitamin D_2_ and high cholesterol diet increased by approximately 3.5 times that of the control rats treated with with high dose of vitamin D_2_ alone. However, the increase in calpain activity was significantly inhibited by CAI, the specific calpain inhibitor (Fig 1A). In addition, oxLDL (25-100mg/L) increased calpain activity in RVSMCs in a concentration dependent manner compared with LDL (25mg/L) (Fig 1B). RVSMCs transfected with siRNA against calpain-1 and treated with oxLDL (50mg/L) showed a decrease in calpain activity compared with that treated with oxLDL (50mg/L) alone. The results suggested that calpain activation might play an important role in vascular calcification. Based on the present result showing that oxLDL (50mg/L) increased calpain activity by approximately 2.5 times compared with LDL and the previous study demonstrating that oxLDL (50mg/L) significantly increased calcium deposition in RVSMCs [28], 50mg/L of oxLDL was chosen in the following *in vitro* experiments.

### CAI reduced vascular calcification in rats with hypercholesterolemia

**Fig 2:**
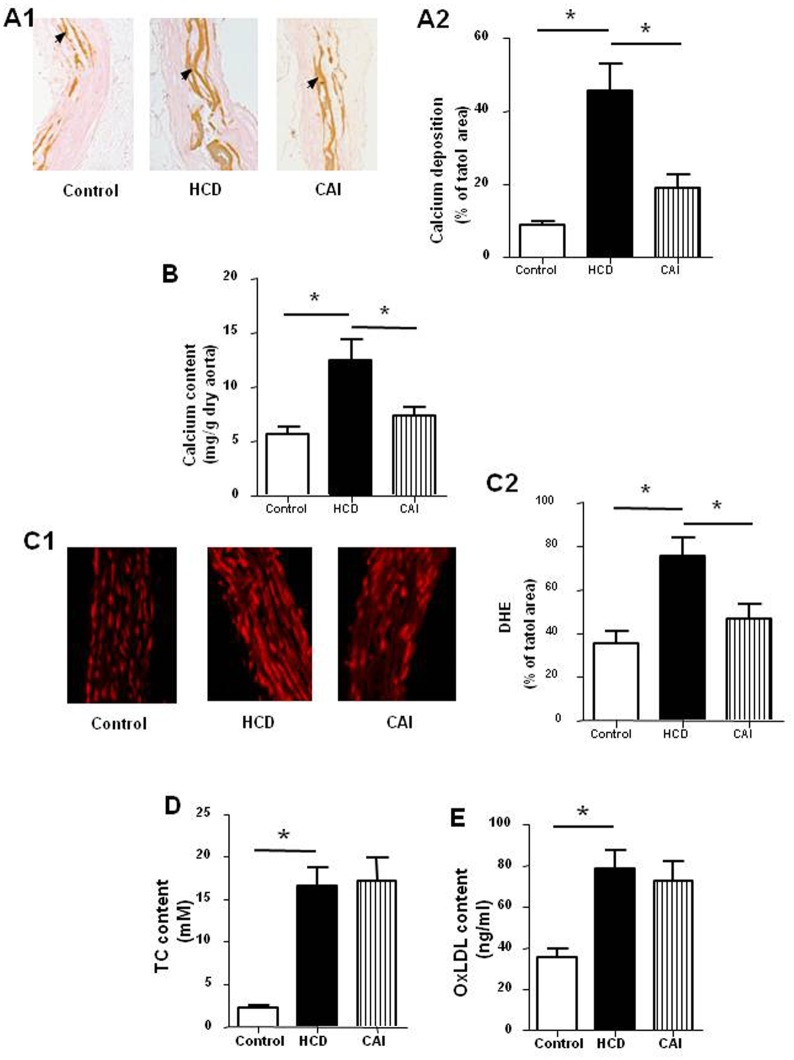
CAI reduced vascular calcification in rats. Rats were treated with high dose of vitamin D_2_ (Control) with or without high cholesterol diet (HCD) in the presence or absence of CAI. The parameters were measured as described in Materials and Methods. A: Calcium deposition in aorta arch in brown yellow color (A1: representative photograph, black arrow indicates the calcium deposition; A2: statistical results); B: Calcium content in aortic tissue; C: DHE in red color indicating superoxide anion (C1: representative photograph; C2: statistical results); D: Serum content of total cholesterol (TC); E: oxLDL content in serum. Results were expressed as mean±S.D. n = 8. *: *P*<0.05 was considered statistically significant.

To test whether calpain activation gets involved in the vascular calcification enhanced by hypercholesterolemia, we observed the effect of CAI, a specific inhibitor of calpain, on vascular calcification. Von Kossa staining of aorta arch showed that the rats treated with both high dose of vitamin D_2_ and high cholesterol diet developed severe vascular calcification demonstrated in brown yellow color compared with control rats treated with high dose of vitamin D_2_ alone (Fig 2A). Similarly, the content of calcium in aortic tissue increased by approximately 2.5 times (Fig 2B) and the over-production of superoxide anion staining with DHE in aortic tissue was found (Fig 2C). In addition, serum contents of TC (Fig 2D) and oxLDL (Fig 2E) increased by approximately 750% and 95% respectively. However, administration of CAI to rats treated with both high dose of vitamin D_2_ and high cholesterol diet alleviated the calcification, decreased the calcium content and attenuated the production of superoxide anion in aortic tissue without affecting the serum levels of TC and oxLDL. The results suggested hypercholesterolemia enhanced the vascular calcification and increased production of superoxide anion, which might be mediated by activation of calpain.

### CAI improved PPi metabolism in rats with hypercholesterolemia

**Fig 3:**
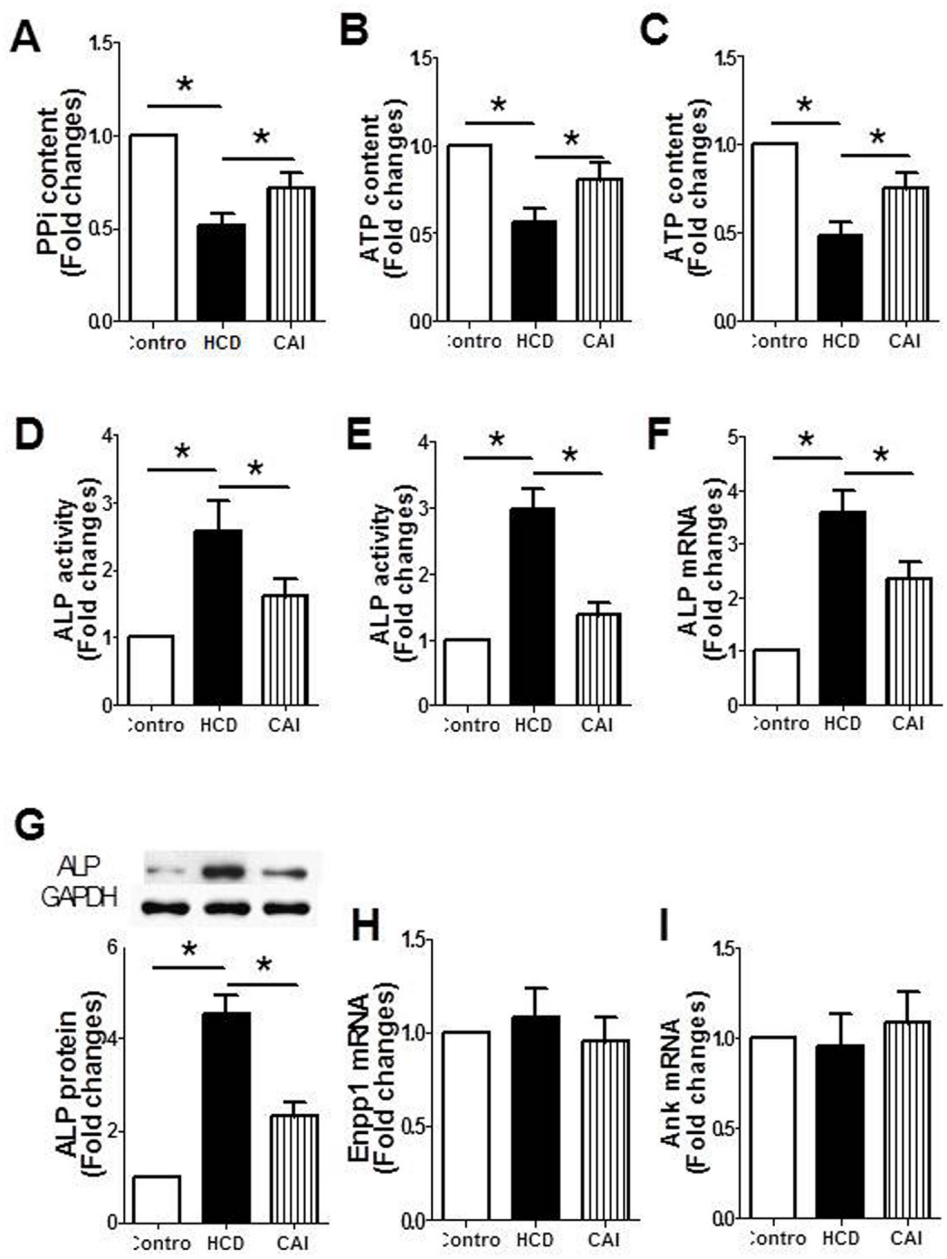
CAI corrected the imbalance in the degradation and synthesis of PPi in rats. Rats were treated with high dose of vitamin D_2_ (Control) with or without high cholesterol diet (HCD) in the presence or absence of CAI. The parameters were measured as described in Materials and Methods. A: PPi content in serum; B: ATP content in serum; C: ATP content in aortic tissue; D: ALP activity in serum; E: ALP activity in aortic tissue; F: ALP mRNA expression in aortic tissue; G: ALP protein expression in aortic tissue (upper: representative photograph; lower: statistical results); H: Enpp1 mRNA expression in aortic tissue; I: Ank mRNA expression in aortic tissue. Results were expressed as mean±S.D. n = 8 for A, B, C, D and E; n = 4 for F, G and I. *: *P*<0.05 was considered statistically significant.

PPi directly blocks *in vitro* and *in vivo* CPD and is therefore a major endogenous inhibitor of vascular calcification [6, 7, 16]. Several key factors are responsible for PPi metabolisms. To further explore the mechanism underlying the calpain-mediated vascular calcification, we tested the effects of CAI on PPi metabolism. The results showed that PPi content in serum (Fig 3A), ATP content in both serum (Fig 3B) and aortic tissue of rats treated with both high dose of vitamin D_2_ and high cholesterol diet (Fig 3C) significantly decreased compared with that of control rats treated with high dose of vitamin D_2_ alone. ALP activity in both serum (Fig 3D) and aortic tissue (Fig 3E), mRNA (Fig 3F) and protein (Fig 3G) expression of ALP in aortic tissue significantly increased. However, these changes were conversely altered by CAI. In addition, both hypercholesterolemia and CAI had no effects on mRNA expression of Enpp1 (Fig 3H), the key enzyme for PPi synthesis, and Ank (Fig 3I), the transporter of ATP, in aortic tissue. The results suggested that CAI alleviated the vascular calcification, which might be related to the improvement of PPi metabolism.

### CAI increased the activity of ATP synthase and protein expression of ATP5D in aortic tissue of rats with hypercholesterolemia

**Fig 4:**
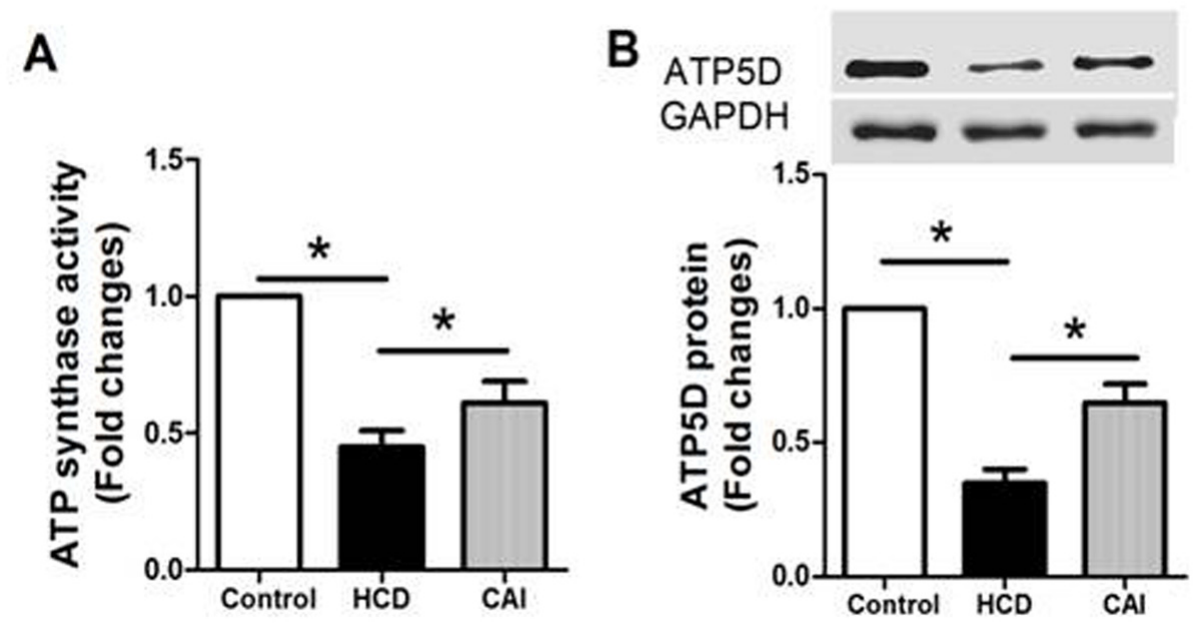
CAI increased the activity of ATP synthase and protein expression of ATP5D in aortic tissue of rats. Rats were treated with high dose of vitamin D_2_ (Control) with or without high cholesterol diet (HCD) in the presence or absence of CAI. The parameters were measured as described in Materials and Methods. A: ATP synthase activity; B: ATP5D protein expression (upper: representative photograph; lower: statistical results). Results were expressed as mean±S.D. n = 8 for A; n = 4 for B. *: *P*<0.05 was considered statistically significant.

ATP5D, δ subunit of the ATP synthase, is one of the main enzymes for synthesis of ATP in mitochondria. The present results showed that both ATP synthase activity (Fig 4A) and protein expression of ATP5D (Fig 4B) in aortic tissue decreased in rats treated with both high dose of vitamin D_2_ and high cholesterol diet compared with that in control rats treated with high dose of vitamin D_2_ alone. However, the decreases in ATP synthase activity and protein expression of ATP5D were inhibited by CAI. The results suggested that improvement of ATP synthase activity and protein expression of ATP5D at least partly contributes to the increase in ATP content by CAI.

### Suppression of calpain-1 inhibited RVSMCs calcification induced by oxLDL

**Fig 5:**
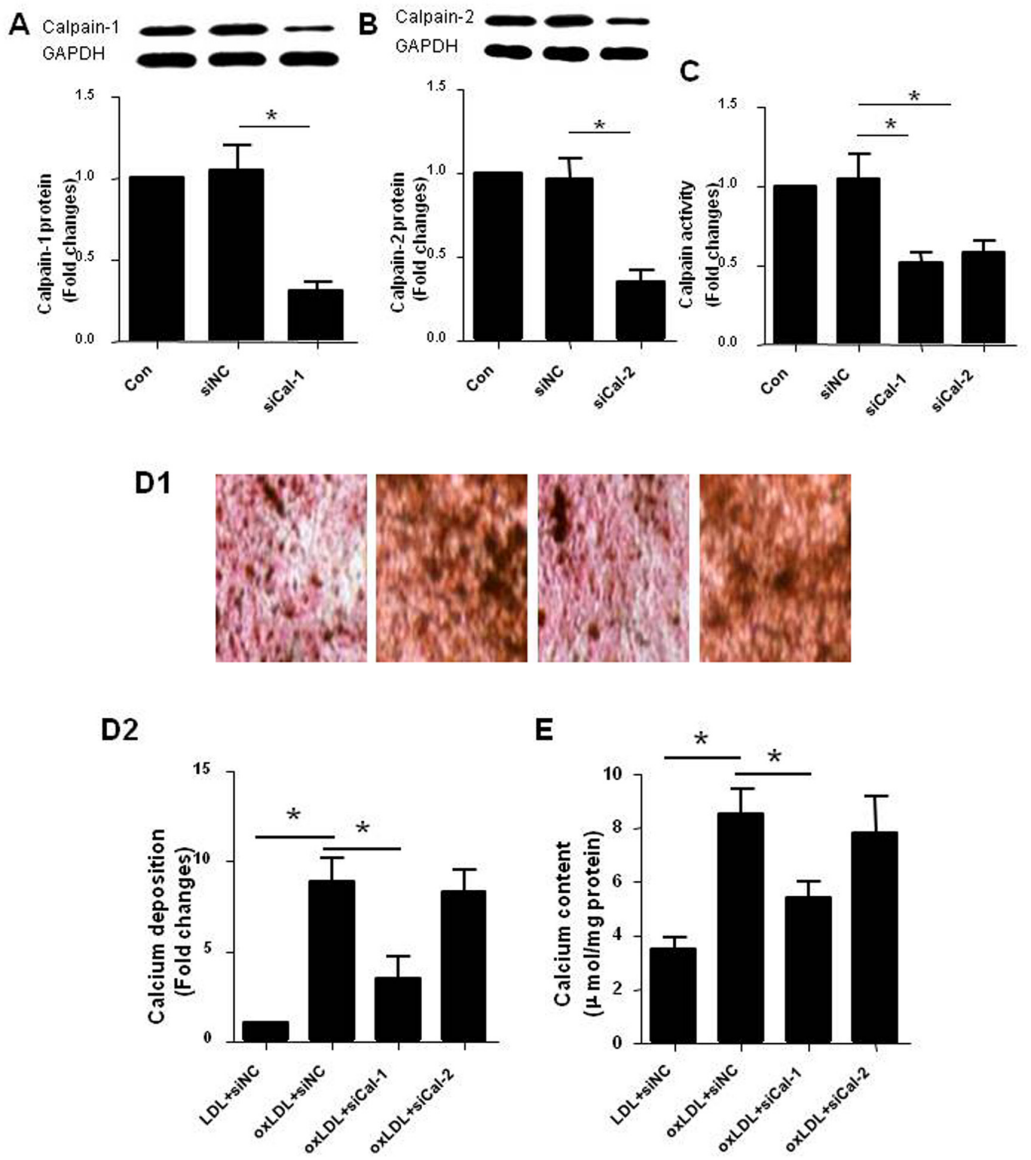
Suppression of calpain-1 inhibited RVSMCs calcification induced by oxLDL. RVSMCs were treated and the parameters were measured as described in Materials and Methods. A: Protein expression of calpain-1 in RVSMCs transfected with siRNA against calpain-1 (upper: representative photograph; lower: statistical results); B: Protein expression of calpain-2 in RVSMCs transfected with siRNA against calpain-2 (upper: representative photograph; lower: statistical results); C: Calpain actitity in RVSMCs transfected with siRNA against calpain-1 and calpain-2 respectively; D: Calcium deposition in RVSMCs in red and black color (D1: representative photograph; D2: statistical results); E: Calcium content in RVSMCs. Results were expressed as mean±S.D. n = 4. *: *P*<0.05 was considered statistically significant

To clarify the role of calpain activation in vascular calcification and to further investigate which subtype(s) of calpain, calpain-1 and/or calpain-2, are (is) responsible for the role, the expression of calpain-1 or calpain-2 in RVSMCs was suppressed using siRNA assay. The effectiveness of suppression was confirmed by the significantly decreased protein expression of calpain-1 (Fig 5A) and calpain-2 (Fig 5B) and calpain activity (Fig 5C) compared with negative control respectively. We then observed the effect of the suppression of calpain-1 or calpain-2 on RVSMCs calcification induced by oxLDL. The results showed that oxLDL induced the calcium deposition (Fig 5D) and increased calcium content (Fig 5E) in RVSMCs, which were inhibited by suppression of calpain-1 rather than calpain-2 expression. The results suggested that calpain-1 rather than calpain-2 plays critical role in the calcification of RVSMCs induced by oxLDL. Therefore, the following experiments were performed using RVSMCs transfected with calpain-1 siRNA.

### Suppression of calpain-1 improved PPi metabolism in RVSMCs treated with oxLDL

**Fig 6:**
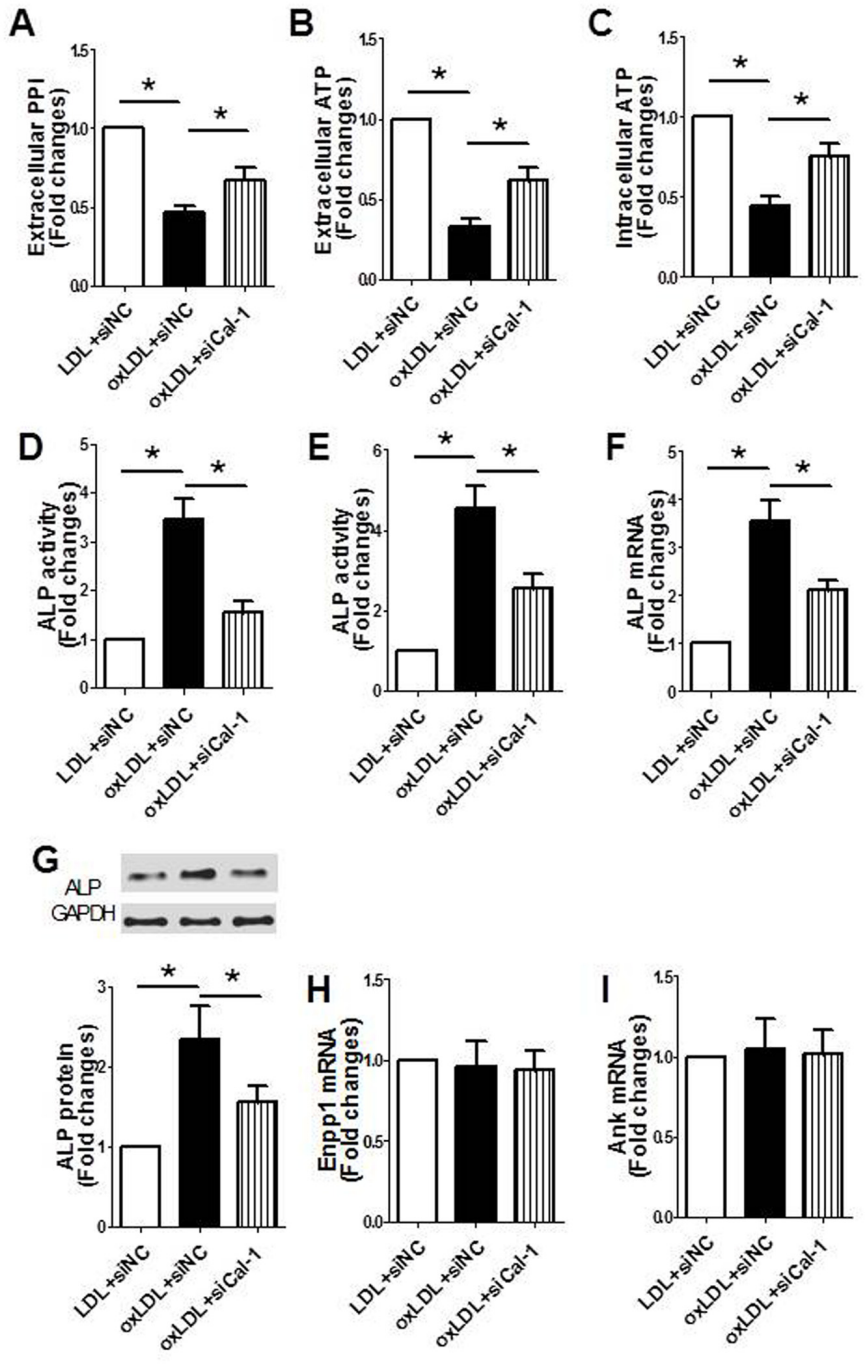
Suppression of calpain-1 corrected the imbalance of PPi metabolism in RVSMCs treated with oxLDL. RVSMCs were treated with or without oxLDL in the presence or absence of calpain-1 siRNA. The parameters were measured as described in Materials and Methods. A: PPi content in medium; B: ATP content in medium; C: ATP content in RVSMCs; D: ALP activity in medium; E: ALP activity in RVSMCs; F: ALP mRNA expression in RVSMCs; G: ALP protein expression in RVSMCs (upper: representative photograph; lower: statistical results); H: Enpp1 mRNA expression in RVSMCs; I: Ank mRNA expression RVSMCs. Results were expressed as mean±S.D. n = 4. *: *P*<0.05 was considered statistically significant.

The results showed that PPi content in medium (Fig 6A), ATP content in both medium (Fig 6B) and RVSMCs (Fig 6C) significantly decreased, while ALP activity in both medium (Fig 6D) and RVSMCs (Fig 6E), ALP mRNA (Fig 6F) and protein expression in RVSMCs (Fig 6G) significantly increased after oxLDL treatment. However, these changes were conversely affected by suppression of calpain-1. In addition, both oxLDL suppression of calpain-1 had no effects on mRNA expression of Enpp1 (Fig 6H) and Ank (Fig 6I). The results suggested that suppression of calpain-1 improves PPi metabolism, which might at least partly explain its anti-calcification effect.

### Suppression of calpain-1 increased the activity of ATP synthase and protein expression of ATP5D in RVSMCs treated with oxLDL

**Fig 7:**
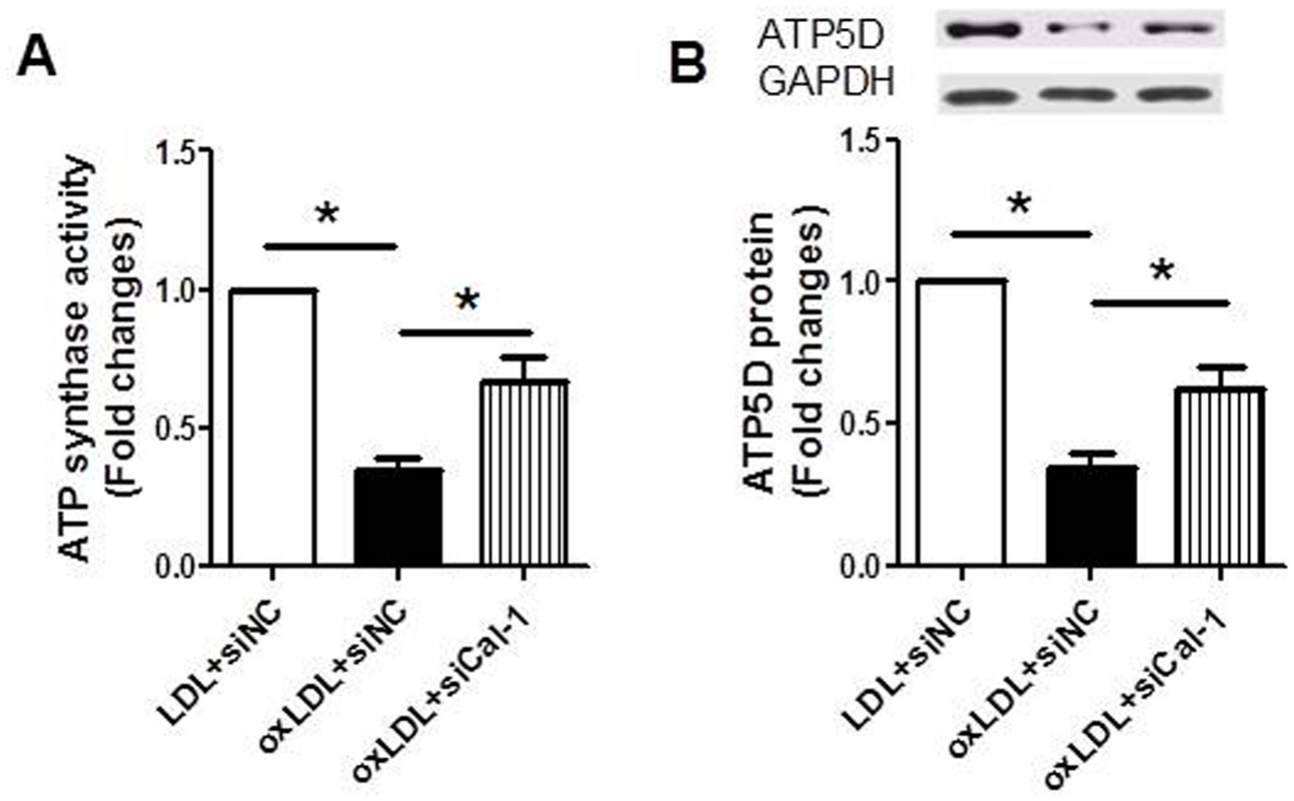
Suppression of calpain-1 increased the activity of ATP synthase and protein expression of ATP5D in RVSMCs treated with oxLDL. RVSMCs were treated with or without oxLDL in the presence or absence of calpain-1 siRNA. The parameters were measured as described in Materials and Methods. A: ATP synthase activity; B: ATP5D protein expression (upper: representative photograph; lower: statistical results). Results were expressed as mean±S.D. n = 4. *: *P*<0.05 was considered statistically significant.

The results showed that the ATP synthase activity (Fig 7A) and protein expression of ATP5D (Fig 7B) in RVSMCs decreased after oxLDL treatment. However, the decreases in activity of ATP synthase and protein expression of ATP5D were inhibited by suppression of calpain-1. The results suggested that improvement of ATP synthase activity and protein expression of ATP5D at least partly contributes to the increase in ATP content by calpain-1 suppression.

### Inhibition of ROS in mitochondria reduced calcification and down-regulated the expression of ALP in RVSMCs treated with oxLDL

**Fig 8:**
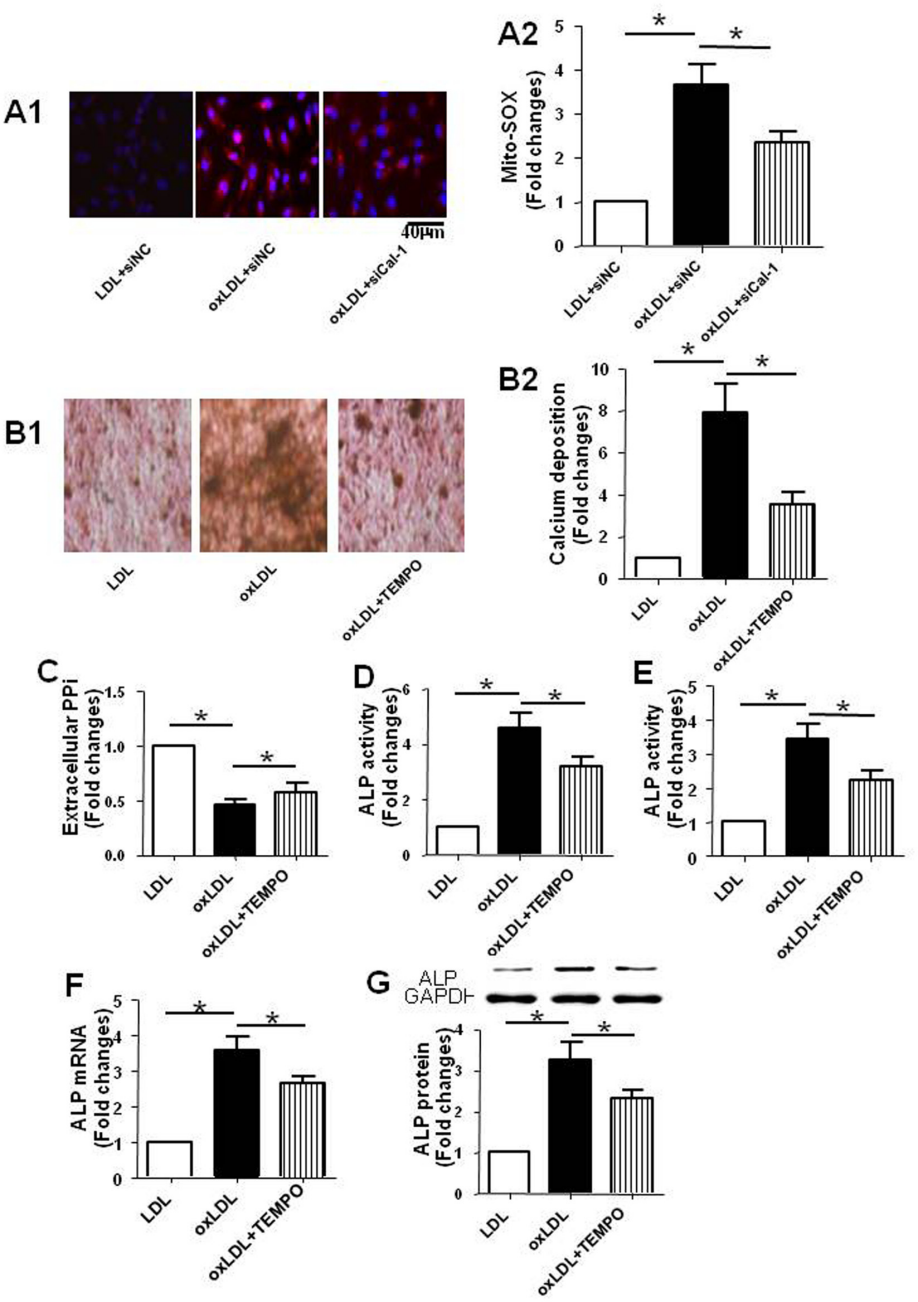
Inhibition of ROS in mitochondria reduced calcification of RVSMCs and regulated the expression of ALP. In one experiment, RVSMCs were treated with or without oxLDL in the presence or absence of calpain-1 siRNA. In the other experiments, RVSMCs were treated with or without oxLDL in the presence or absence of TEMPO. The parameters were measured as described in Materials and Methods. A: Mito-SOX in red color indicating ROS production in mitochondria of RVSMCs (A1: representative photograph; A2: statistical results); B: Calcium deposition in RVSMCs in red and black color (B1: representative photograph; B2: statistical results); C: PPi content in medium; D: ALP activity in medium; E: ALP activity in RVSMCs; F: ALP mRNA expression in RVSMCs; G: ALP protein expression in RVSMCs (upper: representative photograph; lower: statistical results). Results were expressed as mean±S.D. n = 4. *: *P*<0.05 was considered statistically significant.

By using Mito-SOX, the specific indicator for Mito-ROS, we found that Mito-ROS production in RVSMCs treated with oxLDL (Fig 8A) increased compared with that of vehicle treated RVSMCs, which was inhibited by suppression of calpain-1. The results indicated that calpain-1 mediates Mito-ROS production induced by oxLDL. To further investigated the mechanism by which calpain-1 mediates the disorder of PPi metabolism, we tested the roles of Mito-ROS in calcification and regulation of ALP in RVSMCs using mito-TEMPO, a mitochondria-targeted ROS scavenger. The results showed that mito-TEMPO reduced the calcium deposition in RVSMCs treated with oxLDL (Fig 8B), increased the PPi content in medium (Fig 8C), decreased the activity of ALP in medium (Fig 8D) and in RVSMCs (Fig 8E), down-regulated the mRNA (Fig 8F) and protein expression (Fig 8G) of ALP in RVSMCs. Taken together, the results suggested that calpain-1-mediated calcification and disorder of PPi metabolism in RVSMCs caused by oxLDL might be partly attributed to the Mito-ROS induced up-regulation of ALP.

## Discussions

We reported in the previous study that oxLDL accelerates the vascular calcification through over-production of ROS and up-regulation of ALP expression at mRNA and protein levels [28]. The present study showed that CAI, the specific calpain inhibitor, inhibited the vascular calcification in rats with hypercholesterolemia and that siRNA against calpain-1 reduced the calcium deposition in RVSMCs treated with oxLDL. The results suggested that calpain-1 activation mediates the vascular calcification induced by hypercholesterolemia or oxLDL. The findings confirmed and extended the previous study by adding new mechanism by which oxLDL enhances vascular calcification and by shedding new insights into the prevention and treatment of vascular calcification.

The results in the animal experiment showed that hypercholesterolemia enhanced the vascular calcification accompanied by an increase in oxLDL content in serum. Base on the reports that vitamin E, the anti-oxidative vitamin and simvastatin, the HMG CoA inhibitor as well as Tanshinone IIA reduced the vascular calcification through inhibition of the oxLDL formation [28, 35], we assumed that the enhancement of vascular calcification by hypercholesterolemia was mediated by oxLDL. The assumption is supported by the *in vitro* studies, which indicated that oxLDL induced the calcification of VSMCs by stimulating the osteoblastic differentiation of vascular cells [26, 27].

Several studies revealed that calpain-1, the Ca^2+^-sensitive intracellular cysteine protease, plays a critical role in the vascular calcification [23–25]. Over-expression of calpain-1 induces VSMC calcification, which was antagonized by over-expression of calpastatin, a specific inhibitor of calpain-1 [25]. Whether calpain-1 gets involved in the vascular calcification induced by oxLDL has not been reported. The present study suggested that calpain-1 mediates the calcium deposition in aortic tissue of rats with hypercholesterolemia and RVSMCs treated with oxLDL based on the following findings. First, the activity of calpain increased in calcified aorta and RVSMCs, suggesting the potential role of calpain activation in vascular calcification. Second, CAI, the specific inhibitor of calpain, significantly inhibited the vascular calcification in rats with hypercholesterolemia. Third, suppression of the expression of calpain-1 rather than calpain-2 using siRNA assay reduced the calcium deposition in RVSMCs induced by oxLDL. Fourth, both CAI and suppression of calpain-1 expression increased the content of PPi, the endogenous inhibitor of vascular calcification, in rats and RVSMCs respectively. Regarding the possible mechanisms underlying the involvement of calpain-1 in vascular calcification induced by oxLDL, studies showed that during oxLDL-stimulated apoptosis, intracellular Ca^2+^ increases and induces calpain activation, leading to cytochrome c release, apoptosome formation, and caspase activation [17, 29], all are the key factors contributing to the vascular calcification [36, 37]. In addition, calpain-1 was reported to regulate MMP2 activity in VSMCs, consequently inducting the calcium deposition [25]. However, the precise mechanisms by which calpain-1 mediates the calcification caused by oxLDL needs to be further investigated.

It has been shown that PPi directly blocks the *in vitro* and *in vivo* CPD and is therefore a major endogenous inhibitor of vascular calcification [7, 8]. Patients with pseudoxanthoma elasticum (PXE), the ectopic mineralization disorder have a strongly reduced plasma PPi level, explaining their mineralization disorder [38]. Animal studies showed that excessive vascular calcification in progeroid mice is caused by reduced extracellular accumulation of PPi and the calcification is ameliorated on PPi treatment [7]. Therefore, PXE, generalized arterial calcification of infancy, and other ectopic mineralization disorders could be treated with PPi supplementation therapy. ATP, which is synthesized in mitochondria and transported out of the cell by Ank, is the major substrate for PPi synthesis which requires the catalysis of enzyme Enpp1. Degradation of PPi to phosphate is catalyzed by tissue-nonspecific ALP. Our previous study and reports from others showed that ALP is up-regulated in calcified aorta and VSMCs [28, 39–41]. In the present study, we found that the disorders of PPi metabolism appeared in rats with hypercholesterolemia and in ox-LDL treated RVSMCs. The findings are supported by the observations showing that levels of PPi and ATP in serum and culture medium significantly decreased, while ALP activity in serum and culture medium, ALP mRNA and protein expression in aortic tissue and RVSMCs significantly increased. However, no changes in mRNA expressions of Enpp1 and Ank in both aortic tissues and RVSMCs were found, suggesting that the transportation of ATP and process of PPi synthesis are not changed. These results implied that both the reduced ATP level and increased ALP activity and expression might contribute to the decrease in the content of PPi, subsequently reducing its ability to inhibit the vascular calcification. The implication is further verified by the facts that CAI and calpain-1 siRNA improve the PPi metabolism accompanied by the increased level of ATP and decreased activity and expression of ALP. Taken together, the present study suggested that calpain-1 mediates the vascular calcification induced by oxLDL at least partly dependent on disorders of PPi metabolism.

ATP5D, δ subunit of the ATP synthase catalyzes the synthesis of ATP in mitochondria [42–45]. In the present study, we found that the activity and protein expression of ATP5D in aortic tissue of hypercholesterolemia rats and in oxLDL treated RVSMCs significantly decreased. However, the decreases was attenuated by CAI or calpain-1 siRNA respectively. The results suggested that calpain-1-mediated reduction of ATP content might be attributed to the decreases in the activity and protein expression of ATP5D. How calpain-1 reduced the activity and protein expression of ATP5D remains unknown. Studies showed that calpains, the Ca^2+^-sensitive intracellular cysteine proteases, tightly regulate their respective substrates through limited proteolytic cleavage [17, 18]. Therefore, it is assumed that calpain-1 might proteolyze ATP5D, subsequently leading to the reduction of activity and protein expression of ATP5D. This assumption needs to be tested by further investigation.

Dysfunction of ATP synthesis results in over-production of Mito-ROS [46]. Mito-ROS has been shown to regulate the ALP mRNA and protein expression [47]. The present *in vivo* study showed that CAI significantly inhibited the production of superoxide anion, one kind of ROS abundant in mitochondria of aortic tissue, accompanied by the decreased expression of ALP. In addition, the result of the *in vitro* study showed that siRNA calpain-1 reduced the Mito-ROS production in RVSMCs treated with oxLDL, accompanied by down-regulation of ALP expression. These results implied that calpain-1-mediated Mito-ROS production might regulate the expression of ALP, subsequently leading to the disorder of PPi metabolism. To further investigate the roles of Mito-ROS in calpain-1-mediated vascular calcification and the disorder of PPi metabolism, we finally observed the effects of mito-TEMPO, a mitochondria-targeted ROS scavenger, on the calcification and PPi metabolism of RVSMCs treated with oxLDL. The results showed that mito-TEMPO reduced the calcium deposition in RVSMCs treated with oxLDL and corrected the imbalance of PPi as well as decreased the activity, mRNA and protein expression of ALP. Taken together, these results suggested that Mito-ROS gets involved in the calpain-1-mediated up-regulation of ALP of RVSMCs treated with oxLDL, consequently contributing to the calcification of RVSMCs.

In summary, the present study revealed that calpain-1 activation mediates the vascular calcification induced by oxLDL by causing disorders of PPi metabolism, which is an endogenous inhibitor of vascular calcification.
